# Development of an Electrical Resistance Sensor from High Strength Steel for Automotive Applications

**DOI:** 10.3390/s19081956

**Published:** 2019-04-25

**Authors:** Tadeja Kosec, Viljem Kuhar, Andrej Kranjc, Vili Malnarič, Branko Belingar, Andraž Legat

**Affiliations:** 1 Slovenian National Building and Civil Engineering Institute, Dimičeva 12, SI-1000 Ljubljana, Slovenia; viljem.kuhar@zag.si (V.K.); andrej.kranjc@zag.si (A.K.); andraz.legat@zag.si (A.L.); 2 TPV doo Novo Mesto, Kandijska cesta 60, SI-8000 Novo mesto, Slovenia; v.malnaric@tpv.si; 3 DMS Data Measuring Systems doo, Golnik 3, SI-4204 Golnik, Slovenia; branko.belingar@dms.si

**Keywords:** high strength steel, automotive, saline environment, electrical resistance sensor, corrosion

## Abstract

This work focuses on a demonstration of the monitoring of corrosion processes taking place in high strength steel in automotive applications. This is performed by means of a corrosion sensor, which operates as an electrical resistance sensor. It was developed from the same type of material that is used for the high-strength steel parts produced in the automotive industry. Using the sensor, real time corrosion processes can be measured. It is attached to a location inside the vehicle’s engine and is equipped with a data logger, which enables wireless transfer of the measured data. In this study the development, operation, and evaluation of the monitoring process are presented. Corrosion estimation is verified by means of electrochemical methods. A metallographic investigation was included in order to verify the similarity between the microstructural properties of the sensor and those of the as-received high-strength steel sheet.

## 1. Introduction

In recent years new high strength steels have been developed for use in applications in the automotive industry. These special steels retain all the good properties of ordinary steel, but, due to their higher strength, thinner cross-sections of the materials can be used. This reduces the weight of the final product, i.e., the vehicle, which further decreases the carbon footprint.

Different types of high strength steels have been developed, which contain a higher percentage of Mn, Cr and Si, and Al as minor alloying elements. The common properties of these high strength steels are those with appreciable crash behavior, relatively high yield and tensile strength, and an A_80_ elongation that is typically higher than 30%. For this reason structural components that take part in energy absorption during crash situations can have reduced thicknesses, resulting in final lighter weights of vehicles.

Corrosion evaluation of such newly developed steels has not yet been explored, and real time monitoring of such steels in automotive applications has not yet been reported.

In general, the resistometric technique, using electrical resistance probes, has been used relatively frequently for corrosion monitoring in various fields, particularly in the oil and gas industries [1]. In situ DC resistance measurement technique was applied to determine the behavior of the resistance of organic film in a conductive solution [2].

Electrical resistance sensors were utilized to study the atmospheric corrosion of iron and zinc. They were sensitive to corrosion losses of the order of one atomic monolayer [3]. Electrical resistance sensors have been in museums used to monitor corrosiveness of the indoor atmosphere, where sensors were made from pure metals Ag and Cu [4]. A similar comb-like Cu sensor was developed for detection of earlier dew condensation at low relative humidity [5].

The same method of operation of sensors was developed for the monitoring of steel corrosion in concrete [6]. Long term exposure in the field has been reported in the final reports of different European Funded projects [7,8,9]. The modification of such sensors was verified with different protection systems for the monitoring of protected steel structures [10]. The use of electrical resistance (ER) sensors in the field of copper corrosion monitoring, in various media and in bentonite, has been fairly limited [11,12,13]. A similar resistometric method was recently developed to measure the corrosion rate of carbon steel in an anoxic bentonite environment [14].

In previously published papers of our group, which were concerned with a study of copper corrosion in an oxic bentonite/saline groundwater environment inside a bentonite test package, 4 years of measurements were performed on ER sensors [12,13]. The corrosion rate of pure copper was found to decrease from more than 15 µm/year down to 1 µm/year after more than 4 years of exposure and several different evaluation techniques were used to estimate the corrosion rate after the end of the use of the copper corrosion sensor [13]. 

**Figure 1:**
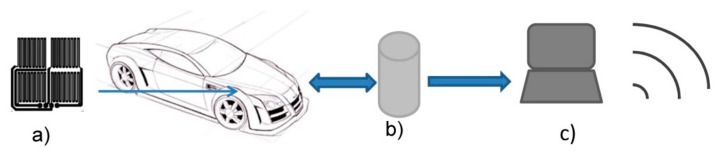
Schematic representation of the method of operation of a sensor for automotive corrosion measurements: (**a**) sensor unit, inserted in vehicle, (**b**) data logger, and (**c**) wireless data aquisition.

In this study, the use of an electrical resistance sensor for the monitoring of high strength steel corrosion in an automotive application was demonstrated. Firstly, the sensor was designed in order to reflect the real corrosion monitoring of a particular new type of steel for an automotive application by wireless data acquisition. The principle is shown in Figure 1. Measurements with an electrical resistance sensor made from high strength steel were performed for 6 months for one sensor, and for 2 months for a second sensor. Electrochemical measurements were also performed on various metal samples in order to compare the results obtained by the use of physical measurements and electrochemical measurements. 

## 2. Materials and Methods

### 2.1. The Metal Sheet

The chemical composition of the 1 mm thick high-strength steel sheet H800 manufactured by Outocompu is given in Table 1. Six of the sensors were fabricated from H800 high strength steel. The evaluation and monitoring of the two sensors are presented in this study. For comparison purposes and the electrochemical measurements, the chemical composition of the currently used complex steel in automotive applications (CP 800) is given in the same table. The mechanical properties of both steels are provided in the same table, which are typical values given in the producer’s technical sheet [15,16].

The 1 mm metal sheet H800 was double-sided and chemically-etched in order to obtain a thinner sheet, while retaining the same microstructural properties. The 1 mm thick steel sheet was etched to a thickness of 250 µm. 

### 2.2. Design of ER Sensor

**Figure 2:**
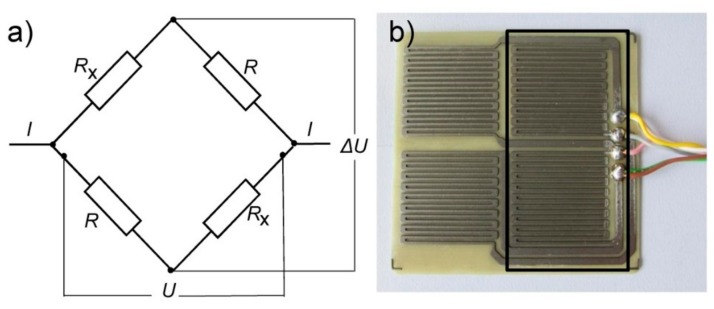
(**a**) Schematic presentation of the corrosion electrical resistance sensor and (**b**) a photo of the sensor, protected with an epoxy coating (rectangle) on the 2 sensor elements with the wiring.

The electrical resistance (ER) sensors were made of high strength steel from a 250 µm thick foil. The 1 mm steel sheets were first chemically etched in order to obtain sensor elements of the form shown in Figure 2b. These sensor elements were then hot-glued onto a glass-fiber resin plate with a thickness of 0.7 mm. The four sensor elements were arranged in a Wheatstone bridge. Two of the sensor elements were exposed, whereas the other 2 sensor elements were protected by a transparent epoxy coating. The two dummy resistors (*R* in Figure 2a) were used for temperature effect compensation. The transparency is deliberate in order to control the state of protected sensors’ elements. The width of the electrical leads in the sensor elements is 0.7 mm. 

### 2.3. Measurements

The ER measurements were performed in order to estimate the corrosion rate of the steels. The technique is based on the fact that a decrease in the thickness of a metallic conductor causes an increase in its electrical resistance [9]. A special conditioning circuit enabling measurement a few micro volts was used with an estimated uncertainty of ±3 µV. The sample rate is adjustable from 1 s to 48 h. For the presented measurements, the sample rate was 6 Sa/h. A decrease in the cross-section of the metallic conductor causes an increase in electric resistance of the metallic conductor:

(1) $$R=\varsigma \cdot \frac{l}{A}$$

where *R* is the electrical resistance of the electric lead in Ω, 𝜍 is the resistivity of the conductor material in Ωm, *l* is length in m, and *A* is the area (cross-section) of the electric lead in m^2^. The resistivity of the conducting material and the length of the electric lead are constant (R·A=const.), therefore

(2) $$\left(R+\Delta R\right)\cdot \left(A-\Delta A\right)=A\cdot R$$

and

(3) $$\Delta A=A\cdot \frac{\Delta R}{R+\Delta R}$$

The ER sensor is composed of four sensor elements arranged in the Wheatstone bridge, where two of the sensor elements are protected (*R*) and two are exposed (*R*_x_). On the assumption that sensors corrode uniformly and that the change of both exposed sensor elements is in the same range as the change of the resistance of exposed element, (Δ*R*) can be written as Δ*R* = *R*_x_ − *R* and the relation between *U,* Δ*U, R*, and Δ*R* can be written as:

(4) $$\frac{\Delta U}{U}=\frac{\Delta R}{2R+\Delta R}$$

Combining Equations (3) and (4) we can get:

(5) $$\Delta d=d_{0}\cdot \frac{2\cdot \frac{\Delta U}{U}}{1+\frac{\Delta U}{U}}$$

where Δ*d* is the change of thickness, *d*_0_ is the expected initial thickness, *U* is the output potential of the bridge, and Δ*U* is the potential drop on the Wheatstone bridge. Then, the remaining thickness (*d*) of a sensor can be written as *d* = *d*_0_ − Δ*d*. The remaining thickness d can be calculated. The remaining thickness (*d*) and change of thickness (Δd) are correlated (d∝−Δd), so the corrosion rate (CR, *v*_corr_) can be defined as the derivative of the additive inverse of the remaining thickness *d* with respect to time *t*:

(6) $$v_{corr}=-\frac{\partial d}{\partial t}$$

The sensitivity of an ER sensor is defined by its thickness, where a smaller thickness affects a shorter service life. The resolution of ER measurements is as good as 0.5 µm/year. More details on ER sensors are given in the paper by our group [12].

For the digital filtering of the results the curves of the remaining thickness of the sensor with the linear curve in the short time interval were first fitted. Afterwards the upper and lower 95% prediction bounds for response values were calculated and all values that were outside of that interval were filtered.

### 2.4. Electrochemical Testing

The testing solution was prepared from analytical grade chemicals. A 0.5 M NaCl solution was prepared. A three-electrode corrosion cell was used for electrochemical tests. The exposed area of the working electrode was 0.785 cm^2^. A saturated calomel electrode (SCE) served as the reference electrode and graphite served as counter electrodes. A Gamry ref 600+ potentiostat/galvanostat was used with Echem analyst software. The electrochemical tests were performed after 1-h stabilization at open circuit potential (OCP). The polarization measurements were performed starting from –0.25 V vs. OCP, and progressed in the anodic direction at a scan rate of 1 mV/s. At least three measurements were performed in order to identify and minimize errors in the electrochemical testing [17].

## 3. Results and Discussion

### 3.1. Metallographic Investigation

**Figure 3:**
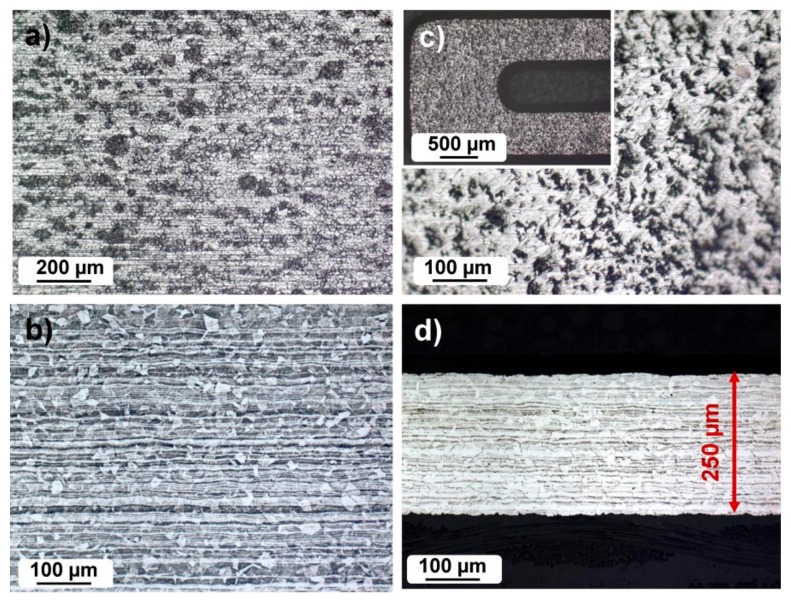
(**a**) The microstructural investigation of high strength steel sheet in top view, as-received; (**b**) the cross-section of the as-received sheet; (**c**) Etched surface, with an inset of the details of the sensor lead, and (**d**) the cross-section.

The microstructural properties of the as-received high strength steel and of the thinned steel sheet are presented in Figure 3. Both the as-received and thinned steel sheets are analyzed in a top view direction and in cross-section. The top view of the as-received high strength steel shows imperfections, rolling lines, and in general a non-homogeneous surface (Figure 3a). The orientation of the crystal grains is uniform in all directions, and the size of the crystal grains is 20–40 µm (Figure 3b). The microstructure of the steel sheet core is completely austenitic. In the core, the austenitic crystalline grains are smaller (Figure 3b) than in the vicinity of the surface. In some crystalline grains, twin boundaries are visible. The top view of the chemically etched surface of the high strength steel shows a relatively rough surface (Figure 3c). The cross-section of the thinned steel sheet is presented in Figure 3d. It can be seen that the microstructure remained unchanged after chemical etching for thinning purposes.

### 3.2. ER corrosion Monitoring Measurements

**Figure 4:**
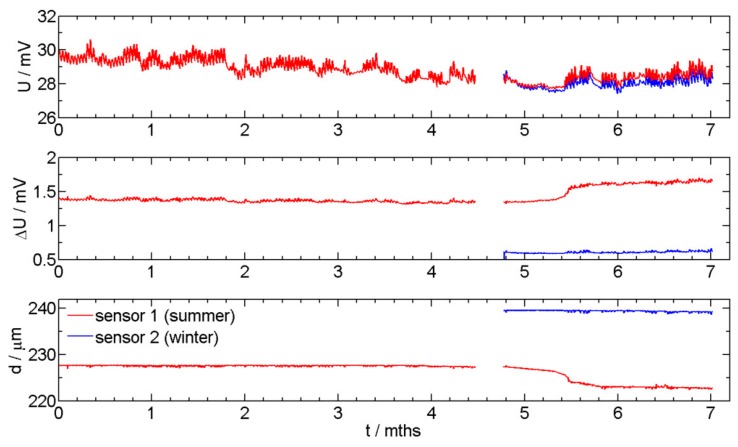
The corrosion sensor readout (the output potential (*U*), the potential drop on the Wheatstone bridge (Δ*U*), the remaining thickness of the sensor (d) exposed for 6 months (red curve), and of the sensor exposed for 2 months (blue curve).

Two newly designed sensors were installed in a vehicle. The installation position is in the inner compartment of a vehicle that is designed from high strength steel. Sensor 1 was initially exposed in summer time (red curve), and sensor 2 was exposed 5 months later in winter time (blue curve). The aim was to monitor the thickness reduction, which is directly correlated to the corrosion rate. Figure 4 presents monitoring of *U*, Δ*U*, and thickness reduction of the readout data for sensors 1 and 2.

**Figure 5:**
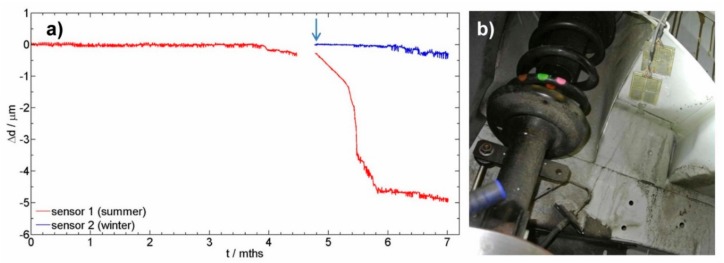
(**a**) The reduction of thickness of the corrosion sensor exposed for 6 months (shown in red), and of the sensor exposed for 2 months (blue curve), and (**b**) position of the 2 sensors exposed for real monitoring in a vehicle.

The values presented in Figure 4 are direct values without adjusting the thickness of the sensor to 250 µm. The first non-corroded sensor has an initial thickness of 228 µm, while the second sensor was initially 239 µm thick. Figure 5a presents the thickness reduction of the two sensors exposed to corrosive environment for two different exposure times. Figure 5b presents the position of the sensors. A filtering process was used in order to remove fluctuations of the recorded data. In the case of sensor 1, which was exposed in the summer time, different intensities of corrosion attack were observed. The steeper the inclination of the curve, the higher the corrosion rate. In the first 4 months of exposure of sensor 1, no visible thickness reduction was observed. In the 5th and 6th month, the winter days resulted is the use of deicing salts, and also a higher humidity was present, thereby thickness reduction is observed. In the last 5 weeks of exposure, out of the total 7 months of the presented exposure, extremely warm and dry days were recorded, which again resulted in a slower corrosion rate (Sensor 1, the red curve). The results of the sensor exposed in winter are also presented in Figure 5 by a blue curve. The start of exposure of sensor 2 is indicated by an arrow in Figure 5a. In the first month of exposure for sensor 2 (months 5 to 6 in Figure 5), the initiation period is present, where no thickness reduction is observed. A slight thickness reduction was observed at the end of the presented exposure.

**Figure 6:**
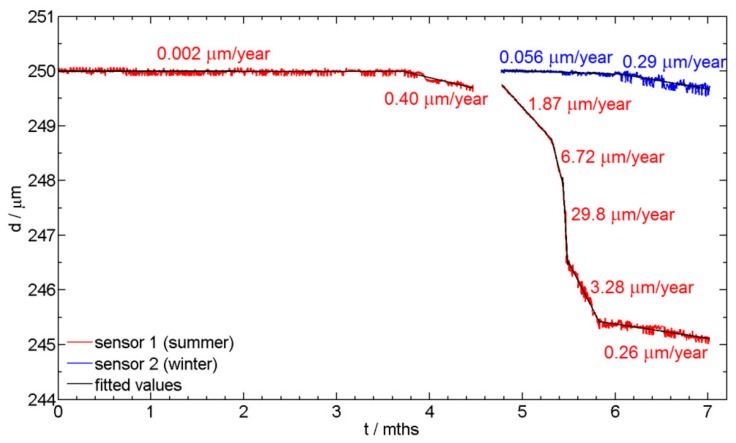
Values of the average corrosion rates in the different sections of the measured data of sensor 1 (summer) and sensor 2 (winter).

The average corrosion rates (in µm/year) at different time sections of the two sensors are presented in Figure 6. The thicknesses of the sensors were adjusted to a value of 250 µm so that both sensors could be compared. The sections of average corrosion rate estimation were chosen according to observe periods of changes in thickness reduction. In the case of sensor 1, which was exposed in summer time, it can be observed that during the first four months of exposure the corrosion rate was just above zero. After four months the corrosion rate increased slightly and it reached its highest rate at the beginning of the sixth month of exposure. The corrosion rate was close to 30 µm/year. Then, it slowed down and in the following dry and warm month it was again very low at 0.26 µm/year. In the case of sensor 2, which was exposed in the winter time, the corrosion rate during the last exposure time was 0.29 µm/year, since the environment at this time of the year is dry and warm. The measured corrosion rates, i.e., for sensors 1 and 2, were very similar, as expected. The different corrosion rates for the two sensors are expected (months between 5 and 6, Figure 6), since the initiation period for the start of the thickness reduction due to the corrosion process is different, due to different corrosion environments for the two sensors in summer and winter time, respectively.

### 3.3. Electrochemical Measurements

**Figure 7:**
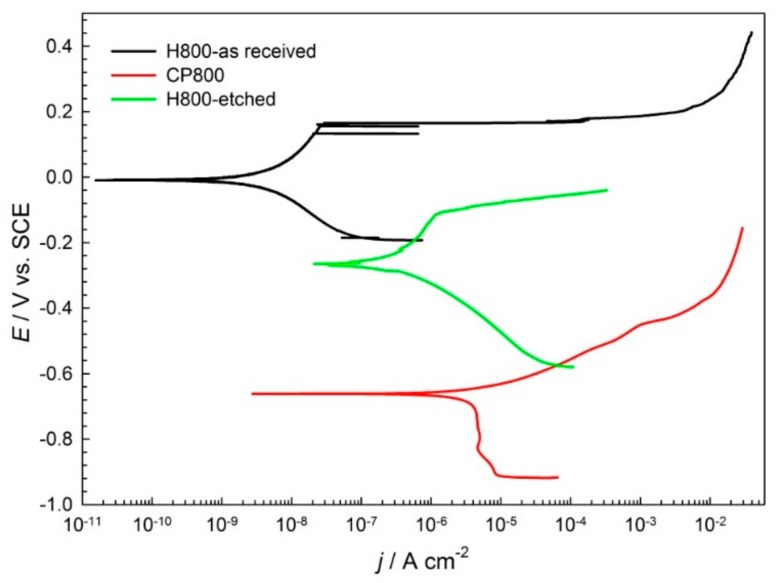
Potentiodynamic scans for the H800-as-received, H800-etched, and CP 800 steels in a 0.5 M NaCl solution, scan rate 1 mV/s.

Potentiodynamic curves were measured in order to study the electrochemical properties of the new high strength steel H800 (as-received and etched surface condition) and the commonly used steel CP-800 in automotive applications. The results are reported in Table 2 and Figure 7.

The high strength steel H800-as-received has a corrosion potential that is quite positive at 0 V vs SCE, whereas the corrosion current density *j*_corr_ is relatively low at 0.018 µA/cm^2^. In the anodic region of the potentiodynamic scan, a passive region is observed with corrosion current densities at around 10 µA/cm^2^. The passive region is limited by the breakdown potential E_b_ at 0.202 V vs SCE. Similarly, the passive region in the anodic scan of potentiodynamic curve is observed for H800-etched steel, while *j*_corr_ is higher at 0.181 µA/cm^2^. Also, corrosion potential *E*_corr_ is lower at −262 mV.

On the other hand, the corrosion potential *E*_corr_ for the CP 800 steel is lower at −0.67 V, and the corrosion current density *j*_corr_ is higher at 4.71 µA/cm^2^. The CP800 material underwent active corrosion, since no passive region was observed in the anodic part of the potentiodynamic curve.

From the current densities measured on the steel electrodes, the corrosion rate was calculated. The corrosion rate CR, in μm/year, was calculated using Faraday’s law [18] as follows

(7) $$CR=3.27\frac{j_{\mathrm{corr}}}{d}\frac{W}{n}$$

where *j*_corr_ is the corrosion current density in µA/cm^2^, 3.27 is a numeric constant in µm g/µAcm year, d is the density in g/cm^3^, and *W* is the atomic weight. For steel, the density and atomic weights are *d* = 7.87 g/cm^3^ and 55.8, respectively; n is the number of electrons required to oxidize an atom.

The measured corrosion current densities translate into 0.21 µm/year for new high strength steel H800, which is in as-received form, while 2.2 µm/year is the corrosion rate for the etched H800 high strength steel. This type of surface condition is similar to the etched thin foils from which sensors were fabricated. The corrosion rate for CP 800 steel is the highest at 55 µm/year (Table 2).

Based on the data obtained from real time measurements and after comparison with the electrochemical measurements, it can be concluded that the values of the corrosion rates, when the sensors are exposed to a very aggressive environment (salinity and humidity), are comparable to those obtained in a solution, when comparing data for high strength steel H800 in etched condition. However, the corrosion rate for the as-received material H800 is lower with its passive film present at the surface, and the corrosion rate for steel CP800 is one order of magnitude higher than for the H800 etched steel. It was shown that the corrosion environment in saline solution is similar to the real environment, where the presence of oxygen, humidity, and salinity are expected and the real time data obtained from online data acquisition and monitoring is similar to those obtained by using electrochemical methods.

## 5. Conclusions

In the present study, the use of an electrical resistance sensor designed from high strength steel was presented for monitoring steel corrosion in an automotive application by real time exposure. The design, monitoring, and evaluation were presented in this study:

(1) The sensor system for automotive application by using ER sensors enabled continuous monitoring of the installed sensors into a vehicle.
(2) The sensors were designed and made from high strength steel used in the automotive industry with dimensions that can fit into the inner parts of a vehicle’s carrosserie.
(3) The thinning of the high strength steel sheet was performed by double-sided chemical etching with the aim of keeping the microstructural properties unchanged.
(4) The corrosion rates estimated from continuous monitoring by ER sensors correlated well with the electrochemical measurements obtained by potentiodynamic tests in a 0.5 M NaCl solution.

## Figures and Tables

**Table 1:** Composition of the H800 and CP800 Steels.

	H800-1 mm	CP800
**Chemical Composition**		
**Fe** [%]	68.0	97.5
**Mn** [%]	16.1	1.41
**Cr** [%]	13.5	0.272
**Al** [%]	0.0097	0.0444
**C** [%]	0.257	0.0713
**Si** [%]	0.375	0.242
**P** [%]	0.0454	0.0174
**S** [%]	0.0019	0.0022
**Mo** [%]	0.0347	0.161
**Ni** [%]	0.216	0.0122
**N** [%]	0.347	0.0097
**Cu** [%]	0.485	0.0095
**Nb** [%]	0.023	0.033
**Ti** [%]	0.0198	0.087
**Mechanical Properties [15,16]**		
*R_p 0,2_* [MPa]	800	680
*R_m_* [MPa]	1000	780
*A_80_* [%]	31	12

**Table 2:** Current density (*j*_corr_) and corrosion rates (CR) estimated from the potentiodynamic curves, scan rate 1 mV/s.

Sample	*E*_corr_ (mV)	*j*_corr_ (µA/cm^2^)	*E*_b_(mV)	CR (µm/year)
H800-as-received	−220	0.0175	203	0.21
H800-etched	−262	0.181	−105	2.1
CP800	−672	4.71	-	55
